# mTOR Activation by PI3K/Akt and ERK Signaling in Short ELF-EMF Exposed Human Keratinocytes

**DOI:** 10.1371/journal.pone.0139644

**Published:** 2015-10-02

**Authors:** Antonia Patruno, Mirko Pesce, Alfredo Grilli, Lorenza Speranza, Sara Franceschelli, Maria Anna De Lutiis, Giovina Vianale, Erica Costantini, Paolo Amerio, Raffaella Muraro, Mario Felaco, Marcella Reale

**Affiliations:** 1 Department of Medicine and Aging Science, University “G. d'Annunzio”, Chieti, Italy; 2 Department of Psychological, Health and Territorial Sciences, University “G. D’Annunzio”, Chieti, Italy; 3 Department of Medical, Oral and Biotechnological Sciences, University “G. D’Annunzio”, Chieti, Italy; University of Pecs Medical School, HUNGARY

## Abstract

Several reports suggest that ELF-EMF exposures interact with biological processes including promotion of cell proliferation. However, the molecular mechanisms by which ELF-EMF controls cell growth are not completely understood. The present study aimed to investigate the effect of ELF-EMF on keratinocytes proliferation and molecular mechanisms involved. Effect of ELF-EMF (50 Hz, 1 mT) on HaCaT cell cycle and cells growth and viability was monitored by FACS analysis and BrdU assay. Gene expression profile by microarray and qRT-PCR validation was performed in HaCaT cells exposed or not to ELF-EMF. mTOR, Akt and MAPKs expressions were evaluated by Western blot analysis. In HaCaT cells, short ELF-EMF exposure modulates distinct patterns of gene expression involved in cell proliferation and in the cell cycle. mTOR activation resulted the main molecular target of ELF-EMF on HaCaT cells. Our data showed the increase of the canonical pathway of mTOR regulation (PI3K/Akt) and activation of ERK signaling pathways. Our results indicate that ELF-EMF selectively modulated the expression of multiple genes related to pivotal biological processes and functions that play a key role in physio-pathological mechanisms such as wound healing.

## Introduction

The interest in the biological interaction of extremely low frequency (ELF) electromagnetic field (EMF) with tissues, has increased due to their possible effect on human health, as well as their potential therapeutic use. Extremely low frequency electromagnetic field (ELF-EMF), with frequencies less than 300 Hz, do not have enough energy to break molecular bonds, nor to cause DNA damage, ionization or even to have thermal effects on cells and tissues [1]. However there have been several evidences that one of the most important physiological effects of ELF-EMF is the promotion of cell proliferation. ELF-EMF effectiveness has been demonstrated by therapeutical applications in tissue regeneration, wound and bone healing [2–6].

Each treatment employing specific characteristics of frequency, modulation and intensity to achieve its efficacy [7, 8].

Cutaneous wound healing is a complex process, in which promotion of migration and proliferation of keratinocytes at the periphery of the wound are steps required for rapid wound closure. When activated by an extracellular stimuli, keratinocytes respond to the trigger through a complex series of cytoplasmic signal transduction pathways. This leads to cytoskeletal reorganization, cell adhesion, as well as the re-programming of cell transcriptional profile [9]. Our group was able to demonstrate that ELF-EMF exposure enhances keratinocyte proliferation and accelerates the switching from the inflammatory phase to the final repair phase, through the modulation of the inflammatory reactions [10, 11]. *In vivo*, continuous exposure to electromagnetic field for 15–30 min for 10 days, or 3 h daily for 8 weeks, positively affects wound healing process [12–14]. Thus, keeping in mind previous studies, we have chosen to study the effect of short-term ELF-EMF exposure on the signaling mechanisms that control the proliferation of the immortalized keratinocyte cell lines (HaCaT), used as research model system standardized, reproducible, and homogenous.

Understanding the molecular effects of ELF-EMF may contribute to develop targeted strategies to promote and facilitate their therapeutic use.

## Materials and Methods

### ELF-EMF exposure system and HaCaT cell culture

The ELF-EMF system used was the same described in previous studies [10, 11, 15]. Briefly, a sinusoidal 50-Hz EMF at a flux density of 1mT (rms) produced by an electromagnetic generator (Agilent Technologies model 33220A, Santa Clara, CA, USA, with stability higher than 1% both in the frequency and in the amplitude) was custom assembled. A current flow of 1.20 A (Ieff) passed through a 160-turn solenoid coil (22 cm length, 6 cm radius, copper wire diameter of 1.25x10^-5^ cm). The generator connected to a power amplifier (NAD electronics Ltd., model 216, London, UK) and the oscilloscope (ISO-TECH model ISR658, Vicenza, Italy) dedicated to monitoring output signals from a Gaussmeter (MG-3D, Walker Scientific Inc., Worcester, MA, USA) were outside the incubator. The achieved MF intensity (1 mT (rms)) was continuously monitored using a Hall-effect probe connected to the Gaussmeter.

Preliminarily, to indicate the warning zones where there is not an optimal spread of EMF and to suggest areas of propagation where the cell culture is uniformly covered by EMF generated, we have simulated the propagation of EMF in a solenoid with the same features of our system. The simulation evidenced that the uniformity of field is of 98%, near the center of the solenoid, where the field lines are parallel to its length. Thus, cell cultures were placed within this region of the solenoid.

Solenoid and control cultures were placed into two identical incubator (HERAcell, Heraeus, Germany) for the same times and conditions. A two channel thermometer (TM–925, Lutron, Coopersburg, PA, USA) was used to record the temperature of the cell culture medium in ELF-EMF exposed and non-exposed samples. Recordered temperature were acquisited and analyzed by Software SW-U801-WIN, and no significant temperature changes were observed associated with application of the ELF-EMF field (∆T = 0.1°C).

Human immortalized non-tumorigenic keratinocyte cell line HaCaT, (Ethnicity: Caucasian; Age: 62 years; gender: Male and tissue: skin) were supplied by CLS Cell Lines Service (Eppelheim, Germany). High-glucose Dulbecco’s modified Eagle’s medium supplemented with 10% fetal bovine serum, 100 U mL^−1^ penicillin and 100 mg mL^−1^ streptomycin was used as a culture medium (Sigma-Aldrich). When cells have reached approximately 80% confluency, HaCaT cells were synchronized by serum-starvation, and then released from cell quiescence by serum re-addition. The synchronized cells from the same flask were seeded in tissue culture plate and incubated at 37°C in a humidified atmosphere and 5% CO_2_ at 37°C for subsequent experiments. HaCaT cells were exposed to 50Hz electromagnetic field at a flux density of 1 mT (rms), while non-exposed control HaCaT cells were placed into an identical incubator in the absence of ELF-EMF stimulation.

### Proliferation assay

The HaCaT cell proliferation was determined by cell count with a hemocytometer and by BrdU cell proliferation assay. All experiments were repeated at least five times in triplicate.

In all experiments performed, cell viability, determined by Trypan blue dye exclusion, was not influenced by field exposure (cells viability > 95%, at the beginning and at the end exposure) and by specific drugs treatment.

### Microarray analysis

One μg RNA of each control and sample was amplified using the “Ammino Allyl MessageAmp^TM^ II aRNA Amplification kit” (Ambion, Austin, TX, USA), able to produce aRNA, containing 5-(3-amminoallyl)-UTP modified nucleotides. The obtained aRNA (5–20 μg) was labelled with Cys3 or Cys5 (GE Healthcare) and hybridized on the array. Cys5-aRNA prepared from each sample of HaCaT cells was mixed with the equivalent amount of Cys3-aRNA from the control cells. A total of 3 technical replicate were performed for each of 3 biological replicate (n = 9). A dye swap hybridization experiment was performed for each biological sample. Signal analysis was assessed as previously described [16]. Briefly, microarrays were dehydrated in ethanol for 2 minutes at room temperature, and dried using compressed air. Microarrays were rehydrated by soaking in prehybridization solution (5x SSPE pH 7, 0.1% SDS, 1% BSA) for 2 hours at 42°C, then were washed in deionized water twice for 1 minute each, and dried by briefly spinning in a centrifuge. Labeled aRNA was denatured at 95°C for 5 minutes, and applied to microarray slide. Microarrays and labeled aRNA were then covered with cover slips (Knittel Glass), placed in Hyb chamber (Gene Machine) and hybridized for 14–16 hours at 42°C. Microarray slides were washed in 2x SSPE, 0.1% SDS at 42°C for 5 minutes, then in 0.1x SSPE, 0.1% SDS once for 2 minutes at room temperature. Slides were then washed three times for 1 minute each at room temperature in 0.1x SSPE, immediately dried by briefly spinning in a centrifuge, and stored in a dark box.

Fluorescent signals were captured by ScanArray 5000 Packard laser scanning (Packard BioChip Technologies, Billerica, MA) and normalized using “ScanArray Express” software.

We used Phalanx Human Onearray chip (Eurogentek, Belgium) with the information of 32,048 features, 30968 detection probes and 1080 control probes.

Microarray data are deposited in the GEO public database (accession number: GSE37833). All data are MIAME compliant.

### qRT-PCR system

Total RNA was extracted using the TRIzol reagent (Life Technologies) according to the manufacturer’s protocol. 3 μg of RNA was reverse transcribed into cDNA using a High Fidelity Superscript, reverse transcriptase kit (Applied Biosystems), according to the manufacturer’s instructions.

We performed quantitative reverse-transcription polymerase chain reaction (qRT-PCR) in an Eppendorf Mastercycler EP Realplex (Eppendorf AG) on individual genes. Each gene-specific primer pair is listed in S1 Table. RPS18 and HPRT were used as internal controls. In this study stability of RPS18 and HPRT not vary in the cells under all conditions. Thus, relative expression of each gene was normalized by RPS18 using the ∆CT method, where ∆CT = C_T(Myc, HnrpA2B1, CDK1, MAP4K4, Eif4G1, Eif2S2, mTOR and RPS6)_-C_T(18S)_ [17, 18]. Predicted cycle threshold values were exported directly into Excel worksheets for analysis. Relative changes in gene expression were determined by the 2^-∆∆CT^ method, were ∆∆Ct = ∆Ct experimental sample -∆Ct calibrator and reported as the difference (n-fold) relative to the value for a calibrator cDNA (control) prepared in parallel with the experimental cDNAs [19]. Relative increase respect to non ELF-exposed cells (mean±SD) are representative of a total of 3 technical replicate performed for each of 3 biological replicate (n = 9).

### Western-Blot

Western blot analysis was performed as described previously [20]. Blots were probed and incubated overnight at 4°C with the rabbit polyclonal IgG anti-AKT (sc–8312), anti-p-AKT (Thr 308, sc–135650), anti-p-p70S6k (sc–7984), anti-p-ERK1 (Thr202 sc–101760), anti-p-p38 (Thr180/Tyr182, sc–101759), mouse monoclonal anti-p-JNK (Thr183/Tyr 185, sc–81502) (Santa Cruz) and rabbit polyclonal IgG anti-mTOR and p-mTOR (S2448, Cell Signaling). A mouse anti-human monoclonal antibody recognizing the human ß-actin (A5441; Sigma-Aldrich) was used as control in all experiments. Data are expressed as mean±SD intensity of optical density.

### Cell cycle analysis

All cells were analyzed using a Beckman Coulter Epics XL flow cytometer. FACS DNA content analysis was carried out by propidium iodide method. The percentage of cell cycle phases (G0/G1, S, and G2/M) was quantified by MultiCycle AV DNA analysis plug-in for FCS Express (De Novo Software, CA, USA).

### Statistical Analysis

All results were expressed as mean ± SD from three independent experiments. For statistical analysis, quantitative data were analyzed by Student t test for unpaired data between exposed to non-exposed cells. Differences are considered significant at p<0.05.

For microarray, Significance Analysis of Microarrays (SAM) software was performed to determine differential mRNA expression. A false discovery rate (FDR) was set at 0% to determine significant genes, with 1000 random class assignment permutations [21].

The networks, functions, and pathways analyses were generated through the use of IPA (Ingenuity Systems, www.ingenuity.com) [22]. Briefly, the Functional Analysis identified the biological functions that were most significant to the data set. Canonical pathways analysis identified the pathways from the IPA library of canonical pathways that were most significant to the data set. Fisher’s exact test was used to calculate a p-value determining the probability of Functions and Pathways associations with the Benjamini-Hochberg multiple testing correction.

IPA Downstream effect analysis predicts the effect of gene expression changes in gene list on biological processes; the regulation z-score algorithm was used to make predictions. The z-score algorithm is designed to reduce the chance that random data will generate significant predictions (significant z-score: z-score ≥ 2 for increased functions, z-score ≤ 2 for decreased functions). The z-score is calculated using only genes that have a known direction of change; these are the genes that display Increases or Decreases in the Findings column. The overlap p-value is calculated using all genes displayed in the Findings column: Increases, Decreases, and Affects.

## Results

### ELF-EMF induces proliferation in human HaCaT keratinocytes

The first aim of this study was to demonstrate the effect of short ELF-EMF exposure on proliferation rates and its correlation with a different cell cycle distribution. Thus, time-dependency experiments were first performed evaluating two different experimental exposure conditions. In one set of experiments, the HaCaT cells were exposed to ELF-EMF only for the first hour, and then grown for the following 23 h outside the ELF-EMF. In the second set, cells were exposed to ELF-EMF for all 24 h of incubation. Cells exposed to ELF-EMF only for the first hour (75±2.7x10^3^ cells) and for 24 h (83±3.1x10^3^ cells) grow more than non-exposed cells (50±1.9 x10^3^ cells) as determined by counting cells using a haemocytometer.

**Fig 1:**
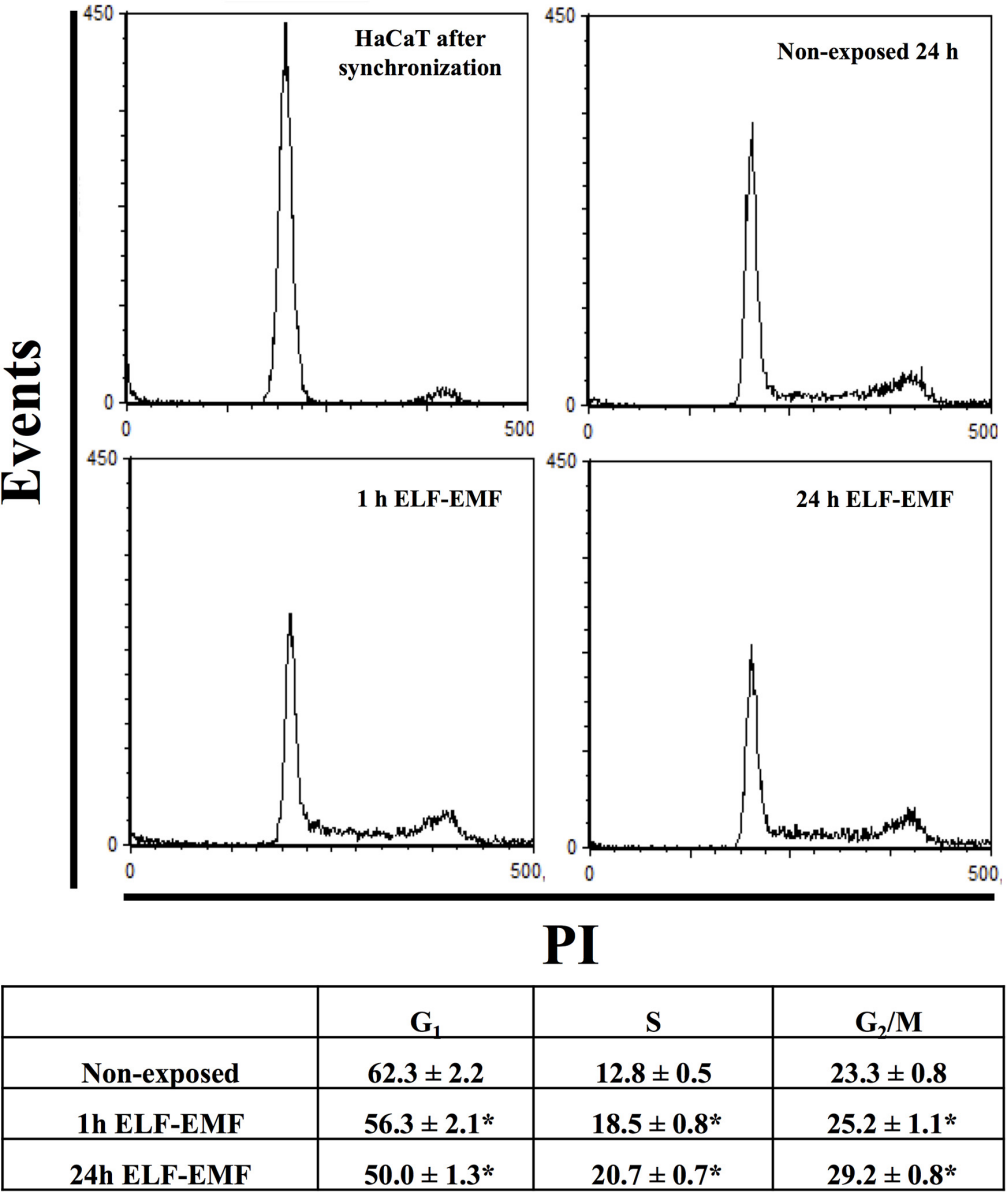
Cell cycle analysis. Flow cytometry cell cycle analysis of HaCaT cells exposed to ELF-EMF (1 mT, 50 Hz) for 1 and 24 h, using PI (40 μg/ml) as probe. Percentage of fluorescent cells for G0/G1, S and G2/M phases were reported in the bottom panel. Mean±SD of three analyses is reported. *p<0.*05 vs* non-exposed cells.

As for the cell cycle distribution, we have observed a significant increases in the percentage of cells in the S phase, in both 1h and 24 h exposed cells compared to non-exposed cells. At the same time both experimental groups showed a significant decrease in the percentage of cells in G0/G1 phase respect to non-exposed cells as showed in Fig 1.

### ELF-EMF modulates PI3K/Akt and MAPK

**Fig 2:**
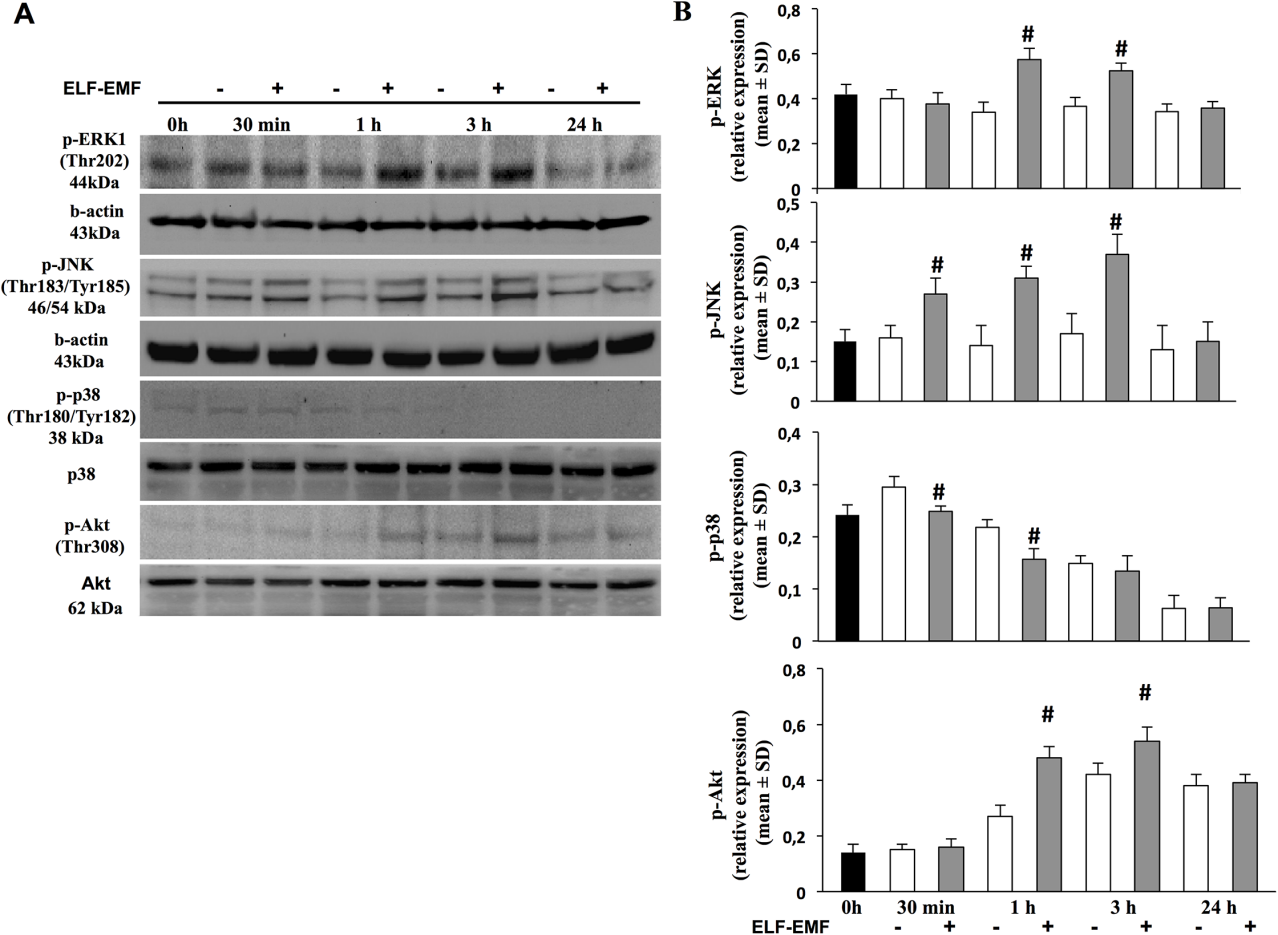
Effect of ELF-EMF exposure on AKT and MAPK activity. A. Representative image of immunoblotting for p-ERK, p-JNK, p-p38 and p-AKT of gels using three separate pools of protein extracted from HaCaT cells time-course exposed to ELF-EMF. B. Averaged band density of p-ERK, p-JNK, p-p38-immunoreactive and p-AKT is expressed as relative expression in both exposed and non-exposed to ELF-EMF (mean±SD; ^#^ *p<0*.*05 vs* time related non-exposed cells).

Since the PI3K/AKT and MAPK pathways have been established as the major proliferative signalling pathways, we investigated their activation in keratinocytes exposed to EMF-ELF [23, 24]. At the end of each EMF-ELF exposure period, 30 min, 1 h, 3 h and 24 h, we analyzed MAPK and the serine-threonine kinase Akt (Thr 308) phosphorylation (Fig 2A). The densitometric analysis of the time-course experiments reported in Fig 2B shows that in non-exposed HaCaT cells, both p-ERK and p-JNK were unchanged at all times-points examined. However, when we examined exposed cells an increased phosphorylation of ERK was observed at 1 h (exposed 0.57±0.04 *vs* non-exposed 0.34±0.05; *p<0*.*05*) and 3 h (exposed 0.52±0.03 *vs* non-exposed 0.36±0.04; *p<0*.*05*) of ELF-EMF exposure. JNK phosphorylation increased after 30 min and peaked after 3 h of ELF-EMF exposure (exposed 0.37±0.06 *vs* non-exposed 0.17±0.05; *p<0*.*05*). After 24 h of exposure, both ERK and JNK phosphorylation decreased to the basal level.

Instead a time-dependent reduction of phosphorylated p38 kinase was observed in both ELF-EMF-exposed and non-exposed HaCaT cells. The cells exposed to ELF-EMF for all exposure times showed the lowest phosphorylation.

The levels of phosphorylation of Akt increased in both exposed or non-exposed HaCaT cells at 1 h (0.48±0.04 *vs* 0.27±0.03, *p<0*.*05*) reaching the maximum after 3 h (0.54±0.05 *vs* 0.42±0.04, *p<0*.*05*) with higher phosphorylation in ELF-EMF exposed cells. After 24 h, levels of pAkt declined, in both exposed and non-exposed HaCaT cells (Fig 2B).

**Fig 3:**
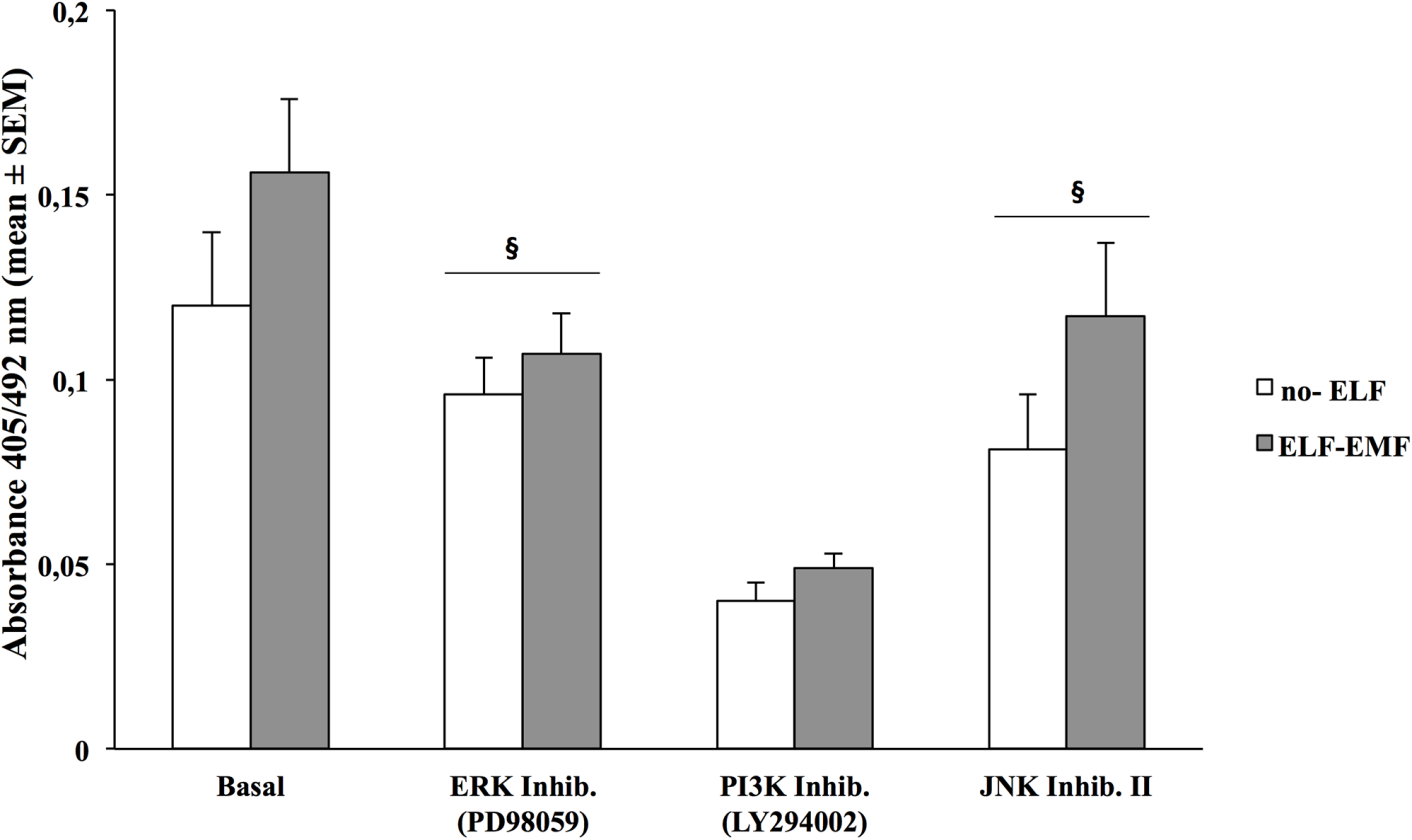
Effects of pharmacological inhibitors on HaCaT cells proliferation. Proliferative rate of HaCaT cells assed by BrdU incorporation assay, after 24 h of incubation in non-exposed and exposed to ELF-EMF only for the first hour. Cells were pre-treated or not with selective inhibitor of ERK kinase activity (PD980559, 1 μM), PI3K (Ly294002, 1 μM), JNK Inhibitor II (20 μM) for 30 min. Each bar represents the mean±SD of three independent experiments performed in triplicate.

To confirm that ELF-EMF-induced kinase activation modulates HaCaT proliferation, we have pre-treated ELF-EMF-exposed HaCaT cells with a specific inhibitor of PI3K/Akt (LY294002), ERK (PD98059) and JNK (JNK Inhibitor II). In Fig 3 we show that in the 1 h only exposed cells, pre-treatment with the three inhibitors reduced cells proliferation, evaluated after 24 h incubation, by 33%, 68,5% and 30% respectively, as compared to un-treated cells (*p<0*.*05*). In non-exposed HaCaT cells proliferation rate was also reduced by 22,5%, 67,7% and 31% (*p<0*.*05*).

### Gene expression profile in HaCaT cells exposed to ELF-EMF

In order to investigate which gene expression was elicited in keratinocytes exposed to ELF-EMF we performed a global gene expression analysis. The Significance Analysis of Microarrays (SAM) analysis identified 153 genes differentially expressed (FDR 0%, Delta = 1.78): 140 genes (12 unmapped) were significantly up-regulated, while 13 genes (1 gene unmapped) were significantly down-regulated after 1 hour of ELF-EMF exposure as reported in S1 File. Ingenuity Pathway Analysis (IPA) revealed that 121 of 153 genes significantly affected by ELF-EMF were pathway eligible.

Analysis of “Downstream Effects” predicted significant decreasing of “Differentiation” (overlap function *p<0*.*01*, z-score <-2) and showed “Proliferation” as the most significant increased function within dataset (overlap function *p<0*.*05*). Also, the function of “Migration” significantly overlapped with gene list (*p<0*.*05*), showing 8 up-regulated transcripts involved in increasing cellular migration. The genes involved in these functions are showed in S2 Table.

To clarify molecular bases of ELF-EMF-induced proliferation, we employed IPA to investigate the main canonical pathways associated with genes list, strictly relating to Growth and Proliferation. The pathway indicated as “Regulation of eIF4 and p70S6K signaling” resulted significantly modulated within “Growth and Proliferation” function (*p<0*.*01*), including up-regulation of translation initiation factors (Eif4G1 and Eif2S2) and ribosomal proteins (RPL8, RPL22 and RPS23).

### qRT-PCR: validation of the microarray data

**Fig 4:**
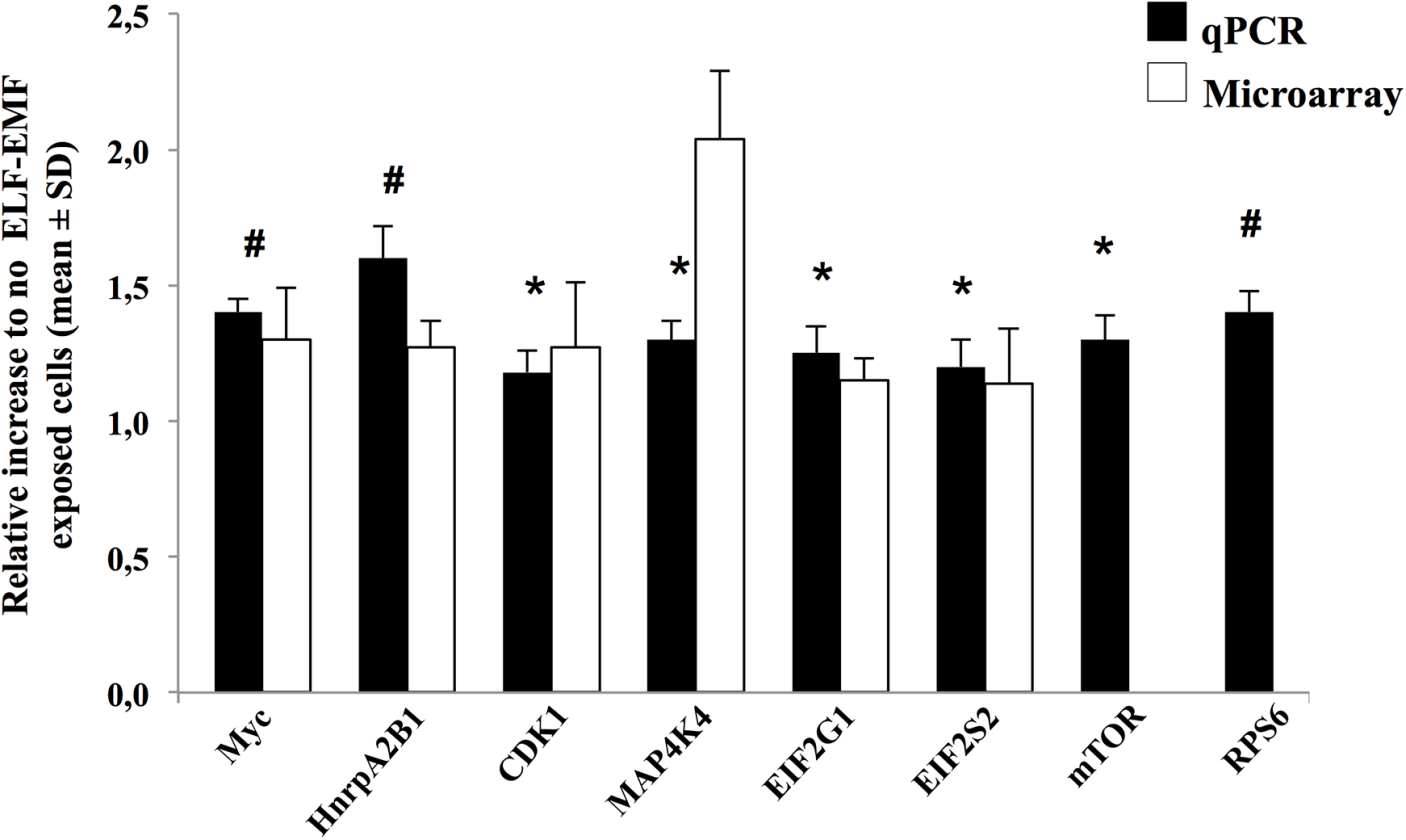
Validation of gene expression using SYBR Green-based qPCR. The following genes Myc, HnrpA2B1, CDK1, MAP4K4, Eif4G1, Eif2S2, mTOR and RPS6 were selected. qPCR data analysis was based on the 2^-△△Ct^ method and RPS18 was used as internal control. The results are plotted as relative increase in not ELF-EMF-exposed cells (*p<0.*05*, #*p<0*.*03*). For all the cDNA templates 1ml were used in a 20μl qPCR amplification system of SYBR Green Real Master Mix Kit as the manufacturer directed. No qPCR products were generated from genomic versus cDNA template. No primer dimers were observed. Melting curve analysis was performed to confirm the purity of the qPCR products. For microarray, Significance Analysis of Microarrays (SAM) software was performed to determine differential mRNA expression. A false discovery rate (FDR) was set at 0% to determine significant genes. mTOR and RPS6 values did not reach the significance and were not showed.

We performed qRT-PCR on single genes in HaCaT cells in the same experimental conditions and exposure used in the microarray assay. Fig 4 showed the eight genes of interest selected: four genes (Myc, HnrpA2B1, CDK1 and MAP4K4) involved in proliferation and migration pathways; and four genes (Eif4G1, Eif2S2, mTOR and RPS6) involved in “Regulation of eIF4 and p70S6K signaling”. qRT-PCR analysis confirmed the microarray results for all genes except mTOR and RPS6 which, although showed the same trend, did not reach the significance.

### ELF-EMF-induced mTOR and p70S6K activation

Since the pathway “Regulation of eIF4 and p70 S6K signaling” resulted significantly modulated in the microarray and IPA analysis, and gene expression patterns do not necessarily correlate with protein levels, we investigated the protein expression of the mammalian target of rapamycin (mTOR) and its effector p70 S6 kinase (p70^S6K^) [25].

**Fig 5:**
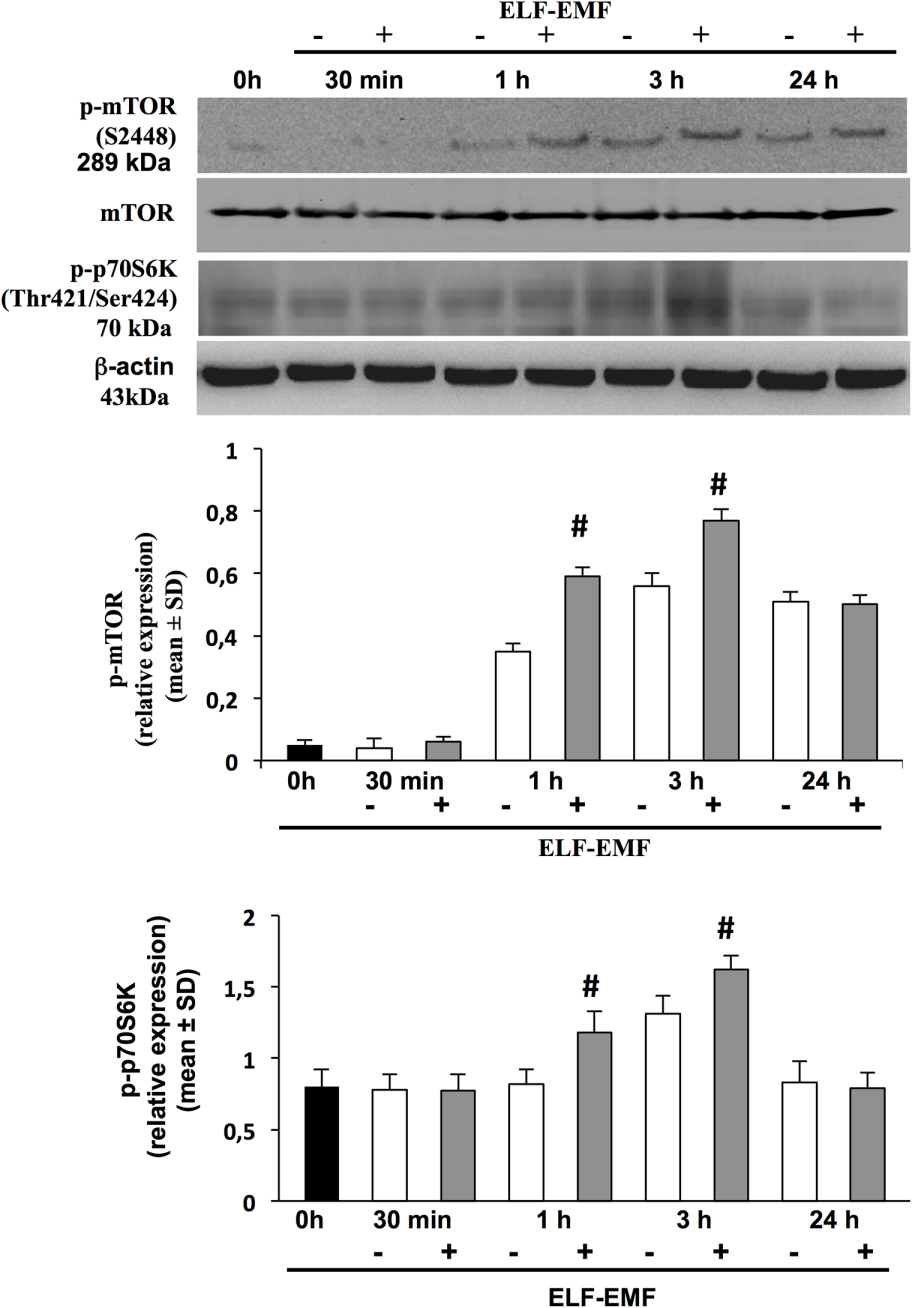
ELF-EMF exposure regulates phosphorilation of mTOR, p70S6k. Expression levels of mTOR and phosphorilated forms for mTOR (p-mTOR) and p70S6k (p-p70S6k) or ß-actin were analyzed by Western blotting, in total lysates derived from HaCaT cells exposed or not to ELF-EMF at 30 min, 1, 3 and 24 h, using specific antibodies. Immunoreactive band were analyzed by densitometry and the relative expressions of p-mTOR to the un-phosphorilated protein, of p-p70S6k to the b-actin are calculated. Each value represents the mean±SD of three different experiments performed in duplicate (^#^ *p< 0*.*05)*.

Therefore, we first performed time-course experiments to analyze the status of activation/phosphorylation of mTOR and its downstream effector p70S6K in both ELF-EMF-exposed (30 min, 1, 3 and 24 h) and in non-exposed HaCaT cells. Western-blot analysis (Fig 5) showed that p-mTOR protein is up-regulated in both exposed and non-exposed cells at 1 h (exposed 0.59±0.03 *vs* non-exposed 0.35±0.02; *p<0*.*05*) and peaked at 3 h (exposed 0.77±0.03 *vs* non-exposed 0.56±0.04; *p< 0*.*05*). Up-regulation of p-P70 S6K-protein level was observed after 1 h only in ELF-EMF-exposed cells (1.18±0.15 *vs* 0.82±0.11; *p<0*.*05*), and at 3 h in both exposed or non-exposed cells (1.62±0.10 *vs* 1.31±0.13; *p<0*.*05*). At 24 h p-mTOR and p-P70 S6K-protein levels declined in both exposed and non-exposed cells.

**Fig 6:**
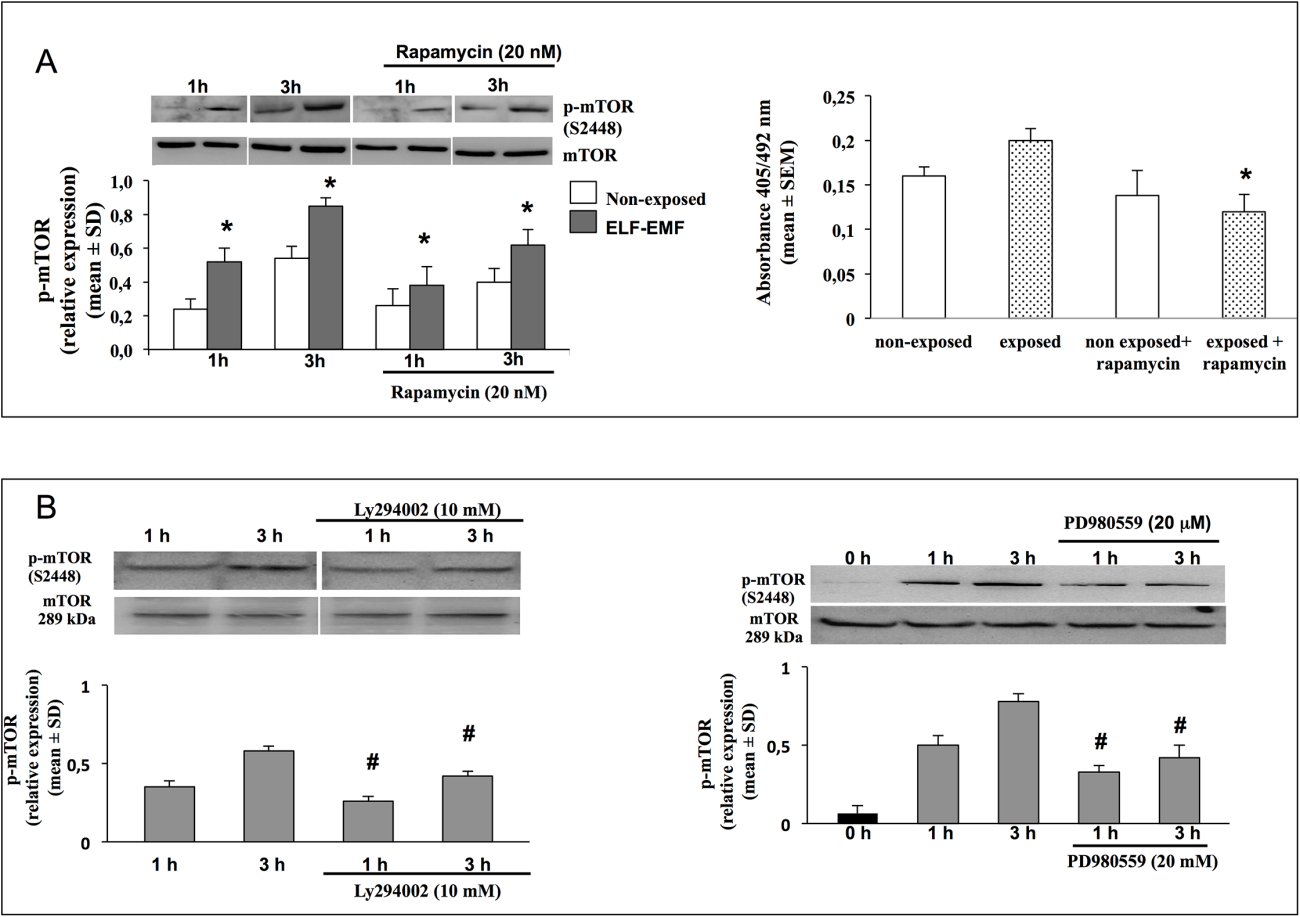
Effects of pharmacological inhibitors on mTOR activation. A. Representative image of immunoblotting for p-mTOR in HaCaT cell non-exposed or exposed for 1 and 3 h to ELF-EMF and treated or not with 20 nM Rapamycin. Data are reported as relative expression of p-mTOR *vs* un-phosphorilated form (mean±SD, n = 3). ^*^ *p<0*.*05* treated cells *vs* cells no-treated with Rapamycin (lef). Proliferative rate of HaCaT cells assed by BrdU incorporation assay, after 24 h of incubation in non-exposed and exposed to ELF-EMF only for the first hour, and treated or not with 20 nM Rapamycin. Each bar represents the mean±SEM of three independent experiments performed in triplicate. ^*^ *p<0*.*05* treated cells *vs* cells no-treated with Rapamycin (right). B. Representative image of immunoblotting for p-mTOR in HaCaT cell non-exposed or exposed for 1 and 3 h to ELF-EMF and pre-treated or not with selective inhibitor of PI3K (Ly294002, 1 μM) and selective inhibitor of ERK kinase activity (PD980559, 1 μM). Data are reported as relative expression of p-mTOR *vs* un-phosphorilated form (mean±SD, n = 6). ^#^ *p<0*.*05* treated cells *vs* cells no-treated with selective inhibitors.

Finally, in order to confirm the role of mTOR in ELF-EMF induced keratinocyte proliferation we used the selective drug rapamycin, to inhibit mTOR activation. Fig 6A shows higher reduction of mTOR activation and cells proliferation in ELF-EMF exposed HaCaT cells compared to non exposed.

We then tested the contribution of Akt and ERK pathways in ELF-EMF-induced mTOR activation. The specific inhibitor of PI3K/Akt (LY294002) in HaCaT cells exposed for 1 h and 3 h, reduced mTOR phosphorylation, more than in non-exposed cells. A significantly reduction of mTOR activation was observed also when ELF-EMF exposed cells were treated with ERK specific inhibitor (PD98059) (Fig 6B). In non-exposed cells, ERK was not modulated at any time and the effect of its specific inhibitor PD98059 was not investigated. These results underline that upon ELF-EMF exposure, both Akt and ERK pathways are involved in mTOR phosphorylation.

## Discussion

The effects of ELF-EMF on cell growth remain a matter of debate, due to discrepancies depending, at least in part, to different experimental conditions like intensity and duration of exposure, modulation of intracellular signals and cell types used [26–29]. Understanding the molecular mechanisms underlying proliferative effect may be relevant in using EMF in clinical settings. Since, the ability of keratinocytes to migrate and proliferate is essential for wound re-epithelialization, we used a well-established human keratinocyte cell line as a model to elucidate the molecular mechanisms of ELF-EMF induced cell proliferation.

Free radical may be mitogenic or cytotoxic depending from levels, efficiency of antioxidant system and cell types. Previous studies showed that ROS generated after short exposure to ELF-EMF play a key role on cell proliferation, as possible initial cellular event. Conversely continuous generation of ROS by long-term ELF-EMF exposures may induce accumulation of DNA damage and slows down cell cycle progression [30, 31].

In this study, we were able to confirm the proliferative effect of short ELF-EMF exposure. HaCaT cells exposed for 1h to 50Hz/1mT showed an increased percentage of cells in the S phase, through a significantly activation of the PI3K, JNK and ERK pathways but not with p38 MAPK activation.

Microarray analysis have showed that in HaCaT cells, ELF-EMF-exposure modulates the expression of genes related to cell proliferation and migration. “Regulation of eIF4 and p70S6K signaling” appeared, in fact, to be the most significantly up-regulated pathway. qRT-PCR confirmed increased mRNA expression levels of selected genes in ELF-EMF exposed cells, and interestingly, also revealed increased levels of mTOR and RPS6, not detected by microarray assays.

Our data are in agreement with the results obtained by Collard and Hinsenkamp (2015). Although they have used another type of stimulation and keratinocyte model, they also highlighted cell proliferation and cell cycle as the main modulated biological processes and their microarray data suggested significant involvement of MAPK and PI3K/Akt upstream mTOR activation [32]. Together with our findings, these observations are interesting for identify a general cellular mechanism induced by ELF.

mTOR protein kinase is a key component of the PI3K/Akt pathway that, through the control of cell proliferation, plays a crucial role in the translational machinery [33]. Interestingly, for the first time our results showed that ELF-EMF induced mTOR activation related to both Akt and ERK pathway. In fact, the PI3K/Akt pathway represent the canonical mechanism for mTOR regulation by the direct phosphorylation on serine 939 and threonine 1462 and inactivation of TSC2, but a novel mechanisms, involving the ERK pathway, but independent from Akt, have been proposed [34–37]. Accumulating evidences indicate that the PI3K-Akt-mTOR signaling pathway may represent a key component of the normal cutaneous healing process, and rates of wound healing are directly dependent by a significantly sustained activation of this pathway. Previous report revealed, by the use of pharmacological and genetically-defined approaches, that the activation of the PI3K-Akt pathway enhances the rate of wound closure dependent on the activation of mTOR [38]. Moreover the activation of mTOR in epithelial cells, by genetic ablation of two of its upstream regulators Pten and Tsc1, accelerate cutaneous wound healing.

It was reported that MAPK and phosphatidylinositol 3-kinase (PI3K), control many cellular functions such as proliferation, survival and differentiation, not acting in isolation but as a network of interconnected signaling [39].

Cross-talk and/or cooperative effects between the PI3K/Akt and Ras/Raf/MEK/ERK1/2 occurs at different levels and exerts cooperative or antagonistic effects depending on external stimuli and cellular background [40–42].

Has been shown that ERK phosphorylates TSC2, predominantly on serine 664, leading to the disruption of the TSC1**–**TSC2 complex, and increasing mRNA translation and mTOR activity [36]. Furthermore, the kinase RSK, a direct downstream substrate of ERK, can also phosphorylate TSC2 on serine 1798 to inhibit the function of TSC1/TSC2 complex [34]. A negative feedback loop has been identified involving Raf/MEK/ERK and PI3K/SGK pathways [43].

Gervais et al. (2006) showed that an over-expressed Akt prevents the nuclear translocation of ERK by stabilizing endogenous PEA15, with a resulting cell proliferation restriction [44].

Our findings provide evidences, in ELF-EMF exposed cells, of the involvement of ERK and Akt pathways in mTOR activation. In fact, this study demonstrated that mTOR activation and consequently cell proliferation were significantly reduced when ERK and Akt pathways were pharmacologically inhibited by PD98059 and LY294002 respectively.

We hypothesize that ELF-EMF may induce a rearrangement of membrane surface proteins and/or activation of membrane receptors (eg. EGFR, Kit, Fms, Flt–3) resulting in Ras/Raf/EK/ERK and PI3K/PTEN/Akt signaling pathways activation, in accord with Soda et al. (2008) and Chiabrera et al. (1984) [45, 46].

This study identifies mTOR as a molecular target of ELF-EMF effect and the Akt and ERK signaling pathways activation as a new model to explain the ELF-EMF-dependent increase of keratinocytes proliferation. In summary, this study identifies the modification of the transcriptomal profile of HaCaT cells after short ELF-EMF exposure. The analysis of the functions of the altered genes draws an interesting picture of the regulation of proliferative signals by PI3K/Akt and ERK signaling pathways resulting in significant phosphorilation of mTOR. These findings provide new molecular insight on ELF-EMF effects, which could be further exploited to support therapeutic approaches for accelerating skin regeneration and wounds closure.

## Supporting Information

S1 File Ingenuity Pathway Analisys gene list. (XLS)

S1 Table Human primers. Primers for selected genes were designed using GeneWorks software (IntelliGenetix, Inc., World Wide Corporation, U.S.A.).
(PDF)

S2 Table Genes involved in differentiation, proliferation and migration. (PDF)
