# Supplementary material for: mTOR Activation by PI3K/Akt and ERK Signaling in Short ELF-EMF Exposed Human Keratinocytes
Source: PLoS One. 2015 Oct 2;10(10):e0139644. doi: 10.1371/journal.pone.0139644 (PMC4592237; doi:10.1371/journal.pone.0139644)
Supplement: S1 Table — Primers for selected genes were designed using GeneWorks software (IntelliGenetix, Inc., World Wide Corporation, U.S.A.). (PDF) [file pone.0139644.s002.pdf]

S1. Table. Human primers

Gene	Forward Primer Sequence [5'-3']	Position in cDNA (EXON)	Reverse Primer Sequence [5'-3']	Position in cDNA	Amplicon Length
RPS18	CTTTGCCATCACTGCCATTAAG	2nd	TCCATCCTTTACATCCTTCTGTC	4th	199bp
HPRT	TGCTGAGGATTTGGAAAGGG	2nd	ACAGAGGGCTACAATGTGATG	3rd	115bp
CDK1	ACAAAGGAACAATTAAACTGGCTG	5th	CTGGAGTTGAGTAACGAGCTG	6th	141bp
EIF4G1	ATTACAAAGCCTCTGAGACCG	21 st	GGTATACTCCACCTTCTGTTCAG	23rd	192bp
EIF2S2	CAGAAAGAGACTACACATACGAGG	5th	AGATGTTTGGGCTGACGATG	7th	199bp
MAP4K4	GTTAAAACGGGTCAGTTGGC	3rd	CCCCACAGAACTCCATAACAAG	5th	196bp
Myc	GCTTCTCTGAAAGGCTCTCC	1st	AGTAGAAATACGGCTGCACC	2nd	155bp
RPS6	TGATGTCCGCCAGTATGTTG	4th	TCTTGGTACGCTGCTTCTTC	5th	149bp
HNRNPA2B1	ACCAGCAACCTTCTAACTACG	9th	GCTCTCATCCTCTCCTATTTATACAG	11th	196bp
mTOR	CAAGAACTCGCTGATCCAAATG	8th	GCTGTACGTTCCTTCTCCTTC	9th	147bp
