# Supplementary material for: mTOR Activation by PI3K/Akt and ERK Signaling in Short ELF-EMF Exposed Human Keratinocytes
Source: PLoS One. 2015 Oct 2;10(10):e0139644. doi: 10.1371/journal.pone.0139644 (PMC4592237; doi:10.1371/journal.pone.0139644)
Supplement: S2 Table — (PDF) [file pone.0139644.s003.pdf]

ID	Genes in dataset	Prediction	Fold Change	Findings
**Differentiation of cells (Z score = - 2,67, overlap p-value 7,74E-03)**				
NM_016542.2	MST4		1,22	Affects
NM_013943.1	CLIC4		1,23	Affects
NM_013436.3	NCKAP1		1,19	Affects
NM_012479.2	YWHAG		1,16	Affects
NM_003345.3	UBE2I	Increased	-1,58	Decreases
NM_002467.2	MYC	Decreased	1,30	Decreases
NM_005638.3	VAMP7	Decreased	1,21	Decreases
NM_012242.1	DKK1	Decreased	1,20	Decreases
NM_003234.1	TFRC	Decreased	1,20	Decreases
NM_002415.1	MIF	Decreased	1,16	Decreases
NM_130469.2	JDP2	Decreased	-1,35	Increases
**Proliferation of cells (Z score = 1,45, overlap p-value 3,36E-02)**				
NM_145686.2	MAP4K4		2,04	Affects
NM_006559.1	KHDRBS1		1,20	Affects
NM_017671.3	FERMT1		1,10	Affects
NM_000358.1	TGFBI		1,09	Affects
NM_003377.3	VEGFB	Increased	1,37	Increases
NM_198793.1	CD47	Increased	1,31	Increases
NM_002467.2	MYC	Increased	1,30	Increases
NM_003144.2	SSR1	Increased	1,28	Increases
NM_001786.2	CDK1	Increased	1,27	Increases
NM_002137.2	HNRNPA2B1	Increased	1,27	Increases
NM_014060.1	MCTS1	Increased	1,23	Increases
NM_001034.1	RRM2	Increased	1,23	Increases
NM_016542.2	MST4	Increased	1,22	Increases
NM_003336.2	UBE2A	Increased	1,21	Increases
NM_003234.1	TFRC	Increased	1,20	Increases
NM_012242.1	DKK1	Increased	1,20	Increases
NM_002415.1	MIF	Increased	1,16	Increases
NM_003592.2	CUL1	Decreased	1,77	Decreases
NM_000268.2	NF2	Decreased	1,24	Decreases
NM_003345.3	UBE2I	Decreased	-1,58	Increases
**Migration of cells (Z score = 0,75, overlap p-value 1,31E-02)**				
NM_198793.1	CD47		1,31	Affects
NM_005978.3	S100A2		1,16	Affects
NM_017671.3	FERMT1		1,10	Affects
NM_003345.3	UBE2I		-1,58	Affects
NM_145686.2	MAP4K4	Increased	2,04	Increases
NM_003377.3	VEGFB	Increased	1,37	Increases
NM_001786.2	CDK1	Increased	1,27	Increases
NM_002137.2	HNRNPA2B1	Increased	1,27	Increases
NM_003472.2	DEK	Increased	1,25	Increases
NM_013943.1	CLIC4	Increased	1,23	Increases
NM_006559.1	KHDRBS1	Increased	1,20	Increases
NM_002415.1	MIF	Increased	1,16	Increases
NM_000268.2	NF2	Decreased	1,24	Decreases
NM_012242.1	DKK1	Decreased	1,20	Decreases
